# 2022 ISCB Accomplishments by a Senior Scientist Award: Ron Shamir

**DOI:** 10.1093/bioinformatics/btac339

**Published:** 2022-06-27

**Authors:** Christina Fogg, Diane Kovats, Martin Vingron

**Affiliations:** Freelance Writer, Kensington, MD, USA; International Society for Computational Biology, Leesburg, VA, USA; Max Planck Institute for Molecular Genetics, Berlin, Germany

Each year ISCB recognizes outstanding contributions by a leader in the fields of computational biology and bioinformatics with the Accomplishments by a Senior Scientist Award. This award is the highest honor conferred by ISCB to a scientist who has made significant contributions to research, education, and service to the field and ISCB. Ron Shamir, Professor of Computer Science at Tel Aviv University in Israel, is being recognized as the 2022 recipient of the ISCB Accomplishments by a Senior Scientist Award. He will receive his award and give a keynote address at the 30^th^ Conference on Intelligent Systems for Molecular Biology (ISMB) in Madison, WI being held from July 10-14, 2022.

*Ron Shamir: Founding Father of Israeli Bioinformatics*


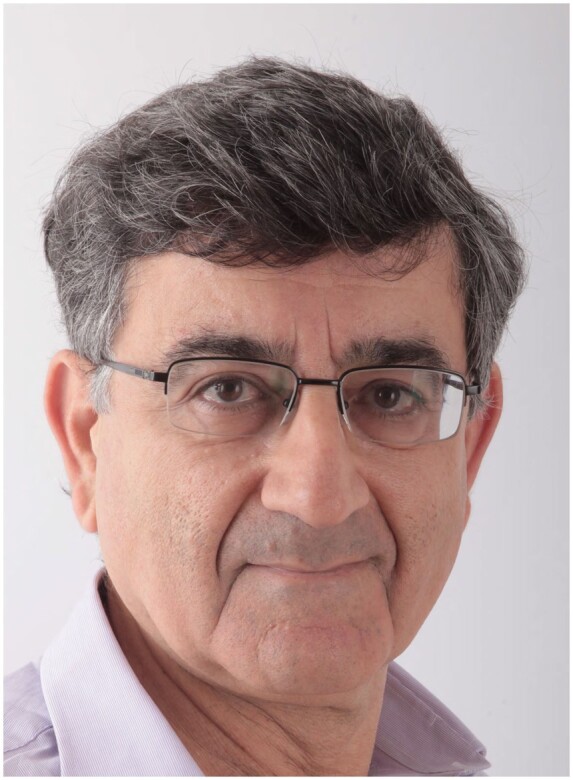


Ron Shamir grew up in Jerusalem, Israel with broad interests in the humanities, science, and mathematics. Shamir was very close to his grandmother who was a pharmacist and had studied chemistry, although he does not recall discussing science with her, and he actually aspired to be an author in his youth. As a high school student at Gymnasia Rehavia in Jerusalem, Shamir became more interested in mathematics, and he recalled, “I had an inspiring math teacher, who really encouraged me. Math problems were like riddles or puzzle-solving challenges. It was fun, and I found that math comes naturally to me.”

Shamir started his BSc in mathematics and physics at Tel Aviv University and then completed his degree at Hebrew University of Jerusalem. He pursued graduate studies in operations research (OR), so he could work on a project that connected math with real-world problems, and enrolled in a PhD program at the University of California-Berkeley (UC Berkeley). Shamir’s PhD research focused on the average case analysis of the Simplex algorithm for linear programming, which fell somewhere between OR and computer science. His PhD advisors at UC Berkeley were Richard (Dick) Karp and Ilan Adler, who he considers pivotal mentors in his career. Shamir said, “Working with them completely transformed my perspective on academic research, and even though I got into grad school with no such intention, I left wishing to try for a career in academia. I have been collaborating with them ever since. Dick has always been an amazing role model to me, and I have learned immensely from him. By sheer coincidence, a few years after my graduation, we both independently got into the emerging field of computational biology and worked on physical mapping algorithms. It was a great pleasure working together in this new field. Dick Karp is one of the giants of theoretical computer science, and his championing and involvement in the young field of computational biology gave it great credibility and helped establish the new area as a bona fide scientific discipline.”

Shamir went on to his first position as a lecturer at Tel Aviv University in the Department of Computer Science where he worked on graph algorithms and optimization. He spent his first sabbatical at Rutgers University, where he worked on temporal reasoning, which is a problem in which event intervals placed along a timeline are subject to various constraints. Gene Lawler had listened to Shamir give a talk about his work, and he recalled, “[Lawler] told me, ‘This is a great model for physical mapping of DNA,’ by replacing time intervals with clones and the timeline with the chromosome. This encounter changed my life. I started reading about DNA and the genome and was hooked. My wife Michal is a biochemist, so I could ask her all the trivial questions about biology, DNA, etc. It was 1990 and the early beginnings of the Human Genome Project, and there was a lot of excitement about the prospects of combining computation and genomics. I dove into this new area, which did not even have a name then, and was not disappointed.” Shamir has gone on to pioneer various algorithmic techniques in genomics, including analysis of microarray data, regulatory motifs, genome rearrangements, and network biology.

Among Shamir’s contributions, he developed elegant algorithms for the analysis of regulatory motifs and protein-protein interactions. Previous approaches have dissected network and similarity data separately, but Shamir and his group developed approaches to analyze these types of data jointly. This led to the discovery of functional modules through the identification of connected networks in the interaction data that exhibited high internal similarity. Shamir continues to be fascinated with topics related to modularity, and he said, “I find myself coming back to the fundamental problem of cluster discovery again and again over the years, and more recently in module discovery based on a combination of similarity and network-based data. This area of study is 100 years old, or 2400 years old, if you start from Aristotle, and is still a lively research area. In a very different direction, I am doing more research on digital medicine in recent years, working on electronic medical records in collaboration with clinicians. This is a tough field. Unlike genomics, where all data is open and well organized, medical data is much more difficult to work with in terms of both data access and data organization. Moreover, physicians are extremely busy work partners and are primarily concerned about treating patients. For them, science only comes second, which makes collaborations more challenging. Nonetheless, this type of research offers a chance to influence disease trajectories and even save lives, so I continue to work in this field.” Most recently, Shamir worked with three of his students and with physicians to analyze COVID-19 inpatient data. They developed a machine learning model for predicting the deterioration of patients 7-30 hours before this process starts and have obtained some promising results. They continue to validate this model by analyzing larger and more diverse datasets, which now include nearly 10,000 patients.

Shamir is well known for making his bioinformatics tools, like the Expander expression analysis suite, readily available to the research community as user-friendly software tools. Some of these tools have been written for a specific project with no intention to be broadly useful, but have become unexpectedly popular, such as the simple UNIX program HYDEN he developed with his student Chaim Linhart to design degenerate primers, which is still downloaded hundreds of times per year. He is a pioneer of bioinformatics education and has been posting bioinformatics lecture notes online since 1997, making his course notes some of the of the most widely used and influential bioinformatics educational materials to date. Shamir has been a deeply committed mentor and advisor throughout his career and encourages his students to choose their own projects, through which he advises and guides their research. He also makes his students draft their own research papers and considers the interaction and joint revision process, which includes corrections and rewrites, to be a key part of their education. Throughout the pandemic, Shamir has been constantly adjusting how his lab interacts, be it through virtual meetings or in-person encounters, and he has tried hard to hold face-to-face meetings with his team as much as possible, in order to help keep their training and development moving forward. Shamir added: “I have been extremely lucky in having incredibly talented, creative and driven students. The interaction with them, and later observing their development into great independent scientists and industry leaders, is extremely gratifying. This is the part of my career I am proudest of.”

Shamir’s research and leadership have been critical to establishing a globally respected Israeli bioinformatics program. He has published over 300 papers, including five with more than 1,000 citations. Shamir established the joint Life Sciences/Computer Science bioinformatics BSc program and founded the Edmond J. Safra Center for Bioinformatics at Tel Aviv University. He was named the Sackler Chair in Bioinformatics in 2003, and his work and service have been recognized by numerous awards, including the Landau National Prize in the Sciences (2010), RECOMB Test of Time Awards (2011 and 2016), Kadar Family Prize for outstanding research, Tel Aviv University (2017), and election as an ACM Fellow by the Association for Computing Machinery (2012) and an ISCB Fellow by the International Society for Computational Biology (2012).

As the 2022 recipient of the ISCB Accomplishments by a Senior Scientist Award, Shamir is deeply honored by this recognition bestowed upon him by his peers, and only wishes his parents were alive to share in this award. He recounted, “As a young faculty member in computer science, I entered an exciting research adventure in an embryonic field that did not even have a name yet. In retrospect, this was a very risky choice before tenure. Seeing how the field developed and matured and being able to help shape it have been great pleasures. This award sums up the path I have taken as a scientist in the past thirty years. I am truly indebted to the society and to the community for it.”

